# In Vivo Model of Osteoarthritis to Compare Allogenic Amniotic Epithelial Stem Cells and Autologous Adipose Derived Cells

**DOI:** 10.3390/biology11050681

**Published:** 2022-04-28

**Authors:** Francesca Veronesi, Milena Fini, Lucia Martini, Paolo Berardinelli, Valentina Russo, Giuseppe Filardo, Berardo Di Matteo, Maurilio Marcacci, Elizaveta Kon

**Affiliations:** 1 Complex Structure of Surgical Sciences and Technologies, IRCCS Istituto Ortopedico Rizzoli, Via Di Barbiano 1/10, 40136 Bologna, Italy; francesca.veronesi@ior.it (F.V.); milena.fini@ior.it (M.F.); 2 Faculty of Bioscience and Agro-Food and Environmental Technology, University of Teramo, Via Balzarini 1, 64100 Teramo, Italy; pberardinelli@unite.it (P.B.); vrusso@unite.it (V.R.); 3 Applied and Translational Research (ATR) Center, IRCCS Istituto Ortopedico Rizzoli, Via Di Barbiano 1/10, 40136 Bologna, Italy; g.filardo@biomec.ior.it; 4 IRCCS Humanitas Research Hospital, Via Manzoni 56, 20089 Milan, Italy; berardo.dimatteo@gmail.com (B.D.M.); maurilio.marcacci@humanitas.it (M.M.); elizaveta.kon@humanitas.it (E.K.); 5 Department of Biomedical Sciences, Humanitas University, Via Rita Levi Montalcini n. 4, 20090 Milan, Italy

**Keywords:** biological treatment, osteoarthritis, animal model, sheep, meniscectomy

## Abstract

**Simple Summary:**

An early resolution of osteoarthritis (OA), through minimally invasive orthobiological solutions, would be important to enable a return to daily and sport activities, and delay prosthesis solutions. No study has yet evaluated amniotic epithelial stem cells (AECs) in OA. They could be considered a valid alternative to adipose derived cells, expanded or concentrated, because they differentiate into three lineages and express mesenchymal and embryonic markers, without a tumorigenic phenotype. The innovative aspects of this study are the comparison of three injective orthobiological treatments, the in vivo use of AECs in OA, and the evaluation of structural and inflammatory fronts of OA for up to six months.

**Abstract:**

The challenge of osteoarthritis (OA) is to find a minimally invasive orthobiological therapy to contrast OA progression, on inflammatory and structural fronts. The aim of the present study is to compare the effects of an intra-articular injection of three orthobiological treatments, autologous culture expanded adipose-derived mesenchymal stromal cells (ADSCs), autologous stromal vascular fraction (SVF) and allogenic culture expanded amniotic epithelial stem cells (AECs), in an animal model of OA. OA was induced in 24 sheep by bilateral lateral meniscectomy and, at 3 and 6 months post-treatment, the results were analyzed with macroscopy, histology, histomorphometry, and biochemistry. All the three treatments showed better results than control (injection of NaCl), but SVF and AECs showed superiority over ADSCs, because they induced higher cartilage regeneration and lower inflammation. SVF showed better results than AECs at 3 and 6 months. To conclude, SVF seems to be more favorable than the other biological options, because it is easily obtained and rapidly used after harvesting, with good healing potential. AECs cause no discomfort and could be also considered for the treatment of OA joints.

## 1. Introduction

Osteoarthritis (OA) has been studied for years, but a consensus on its treatment with orthobiology has not been reached, and, therefore, different local or systemic therapeutic strategies are applied in clinical practice. The purpose of most therapy regimens is to restore joint homeostasis, to reduce joint inflammation, and to improve the functional framework. However, no therapeutic option can improve all these aspects in OA, nor counteract the evolution of degeneration [1,2]. Therefore, a definitive and standardized conservative care does not exist, and the invasive surgical procedures employed to avoid or postpone total knee substitution provide only unpredictable and incomplete results [3]. Finally, there is a lack of studies that compare different stem cell sources in relevant OA models.

Preclinical research in OA is increasingly focusing on minimally invasive biological therapeutic solutions to improve the articular surface and offer a rapid return to daily activities in mild OA. Among these therapies, most are injectables such as growth factors (GFs) or cells-based treatments [4]. In this context, mesenchymal stromal cells (MSCs) have taken hold due to their proliferative, immunomodulatory, and anti-inflammatory properties through the production of several paracrine factors. In addition, the ability to stimulate the synthesis of cartilaginous matrix and to diminish the degradative enzymatic activity are also appreciated [5]. 

Among the different tissues where it is possible to isolate MSCs, adipose tissue shows advantages over bone marrow: it is more easily isolated, with lower patient discomfort, and possesses a higher number of MSCs (5% of nucleated cells). Adipose derived mesenchymal stromal cells (ADSCs) have higher genetic stability, proliferation, differentiation and immunoregulatory abilities, with lower senescence than bone marrow mesenchymal stromal cells (BMSCs) [6]. The regenerative and anti-inflammatory effects of culture expanded ADSCs in OA are well documented in both preclinical and clinical studies [7,8,9]. However, they require a procedure with two surgical steps (one for the cell harvesting and one for the infiltration, after culture expansion), long time (nearly 2 weeks) and specialized laboratories (cell factories). Therefore, to limit the disadvantages associated with these two steps, a one-step procedure has been proposed with the use of the stromal vascular fraction (SVF). SVF has been employed in animal models of OA, in mice and sheep [6,10], and in clinical studies in patients affected by knee OA [11,12,13]. SVF includes ADSCs in addition to other cells, such as progenitor cells, endothelial cells, fibroblasts, monocytes, macrophages, immune cells, muscle cells, pericytes, CD34+ cells, GFs, and few adipocytes and stromal components, which constitute the so-called “niche” [14]. Another MSC source is emerging as a new solution, easy to obtain, with no discomfort for the patient and with a high capacity for regeneration and inflammation reduction: the placental tissue. Cells derived from the inner membrane of the placenta, the amniotic epithelial cells (AECs), possess a high plasticity level, can differentiate towards different lineages, and are immunomodulatory [15,16]. In bone regeneration and in the musculoskeletal field, AECs are mostly studied in vivo for the treatment of alveolar bone defects [17,18] or tendinopathies [19,20,21], showing promising results. Regarding cartilage, only two in vitro studies showed that human AECs improved cartilage defects in cartilage explants treated with human amniotic membrane and AECs [22,23]. By also considering the lack of relevant studies comparing different MSC sources in OA, this in vivo study aimed to compare the potential of these three cell-based injective treatments (SVF, ADSCs and AECs) in a relevant large animal model of OA.

## 2. Materials and Methods

Animal allocation into the groups and treatments were conducted not blinded for investigators, while outcome assessment and data analysis were conducted blinded for the analyzers.

### 2.1. Study Design 

**Figure 1:**
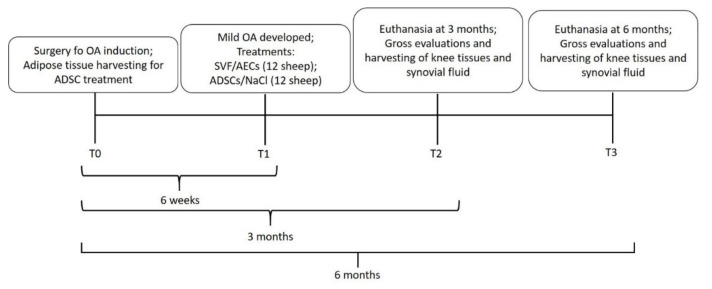
Schematic representation of the timeline of the study.

Figure 1 outlines the timeline of the study. 

At T0, a bilateral lateral meniscectomy was performed and, after 6 weeks (T1), mild to moderate OA was developed [24], and one intra-articular (i.a.) injection of each treatment (1 mL) was administered as follows:

(1) autologous culture expanded ADSCs (12 joints);
(2) allogenic culture expanded AECs (12 joints);
(3) autologous SVF (12 joints);
(4) 0.9% NaCl (Control) (12 joints).

### 2.2. Treatments

The administration of the treatments was assigned randomly, but the randomization did not interest the whole group of animals to limit the number of anesthesia for the animals. It was, therefore, preferable to assign random associations of treatments that did not involve more than one anesthesia per animal by changing only the implant side (right limb or left limb) because the association of SVF and ADSCs in the same animal would imply two anesthesia in two months (OA induction and adipose sampling) in the same animal. Therefore, randomization was performed as follows:

(1) A1 (right: SVF, left: AECs);
(2) A2 (left: SVF, right: AECs);
(3) B1 (right: ADSCs, left: 0.9% NaCl);
(4) B2 (left: ADSCs, right: 0.9% NaCl).

ADSC treatment: At T0, abdominal adipose tissue (25 ± 5 g was harvested and sent to the laboratory. It was digested with 0.075% collagenase II (Sigma Aldrich, St. Louis, MO, USA). The enzymatic reaction was stopped by the addition of complete medium [Dulbecco’s modified Eagle’s medium (DMEM) supplemented with 10% fetal bovine serum (FBS) (Lonza, Verviers, Belgium), 100 U/mL penicillin, 100 mg/mL streptomycin (Gibco, Invitrogen, Carlsbad, CA, USA) and 5 mg/mL plasmocin (Invivogen, San Diego, CA, USA). Cells were centrifuged and the nucleated ones were then seeded in complete medium. At subconfluence at P2, the adherent cells were detached, counted and 2.5 × 10^6^ cells/mL were injected 6 weeks after (T1). 

AEC treatment: At T1, AECs were previously in vitro expanded for 3 passages in an Eagle-α modification medium (α-MEM) supplemented with 20% fetal calf serum (FCS), 1% ultraglutamine and 1% penicillin/streptomycin without any growth factors, in the Faculty of Biosciences and Agri-Food and Environmental Technologies (University of Teramo), before delivering them to the surgery room. An amount of 2.5 × 10^6^ cells/mL was injected, according to Canciello et al. [25].

SVF treatment: At T1, the autologous abdominal adipose tissue (25 ± 5 g) was harvested and sent to the laboratory. It was digested with 0.075% collagenase II for 1 h and then the reaction was stopped with complete medium. Cells were centrifuged and 1 mL of suspension was injected into the joint. The total procedure took nearly 1 h and 30 min.

NaCl control: at T1, 1 mL of sterile NaCl was injected into the joint. 

After 3 (T2) and 6 (T3) months following treatment, animals were pharmacologically euthanized with intravenous administration of 20 mL m-butamide, mebenzonium iodine, and tetracaine chloride Tanax^®^ (Intervet-Italia S.r.l., Milan, Italy) under deep general anesthesia. 

Synovial fluid was harvested with a syringe, the joints were carefully opened, the synovial membrane was harvested, and the macroscopic appearance of tibial plateau and femoral condyles was evaluated. 

### 2.3. Surgical Procedure

Twenty-four adult Bergamasca x Massese sheep (47 ± 5 kg) (Pancaldi, Budrio Bologna, Italy) were used in accordance with 2007/526/CE Recommendations [26] and under veterinary control. The animals were housed in single boxes at room temperature (T), relative humidity (RH) of 22 ± 1 °C and 50 ± 10% RH and ventilation of 12 air changes per hour. Animals were fed with a standard pellet diet (Mucedola, Milan, Italy), clover and water ad libitum. 

After 7 days of quarantine, in all animals, under general anesthesia, bilateral lateral meniscectomy was performed, according to Delling et al. [24]. 

More precisely, the day of surgery (T0), an intramuscular injection of 10 mg/kg ketamine (Imalgene1000 Merial Italia S.p.A., Italy), of 0.3 mg/kg xylazine (Rompun, Bayer, Italy), and a subcutaneous injection of 0.0125 mg/kg atropine sulphate were used for premedication. Intravenous injection of sodium thiopental in a 2.5% solution (6 mg/kg, Penthotal sodium, MSD, Intervet production S.r.l., Aprilia, Latina, Italy) induced general anesthesia, maintained through 2–3% sevofluorane administration (Sevoflurane Baxter 100%, Baxter SpA, Italy) in O_2_/air 60%/40%, 7.5 L/min (Servo Ventilator, 900D; Siemens, Prittriching, Germany). 

With the animals in dorsal recumbence, both legs were prepared for aseptic surgery. In both legs, after a vertical skin incision, the cranial and lateral attachments of the lateral meniscus were transected, removing completely the meniscus. The cartilage was not damaged. All surgeries were performed by the same veterinary surgeon. The postoperative therapy consisted of: cephalosporin 1 g per day for 5 days and analgesics (metamizole sodium 40 mg/kg/die intramuscular, for 3 days; ropivacaine 7.5 mg/mL intramuscular, and Fentanyl 50 µg/72 transdermic patch). The sheep were allowed to fully weight bear and to move freely without any constraints. All animals underwent veterinary examination in the post-surgery period and during the follow-ups with a specific check list that evaluated symptoms.

### 2.4. Evaluations

The knees of all animals were used for each analysis (n = 6 for each group and experimental times). 

#### ADSC and AEC Characterization

Before cell injection into knee joint, to be sure that the cells isolated from the adipose tissue were really ADSCs, they were characterized for their epitope through fluorescein isothiocyanate (FITC)-conjugated antibody against CD31, CD45, CD34, CD44, CD73, CD90, and CD105, with FITC-conjugated nonspecific IgG used as isotype control (BioLegend, San Diego, CA, USA).

For CFU-F assay, 200 ADSCs/cm^2^ were seeded in T25 flasks and cultured for 10 days, fixed with 10% formalin, and stained with 0.1% toluidine blue in 1% paraformaldehyde (PFA) for 1 h. The aggregates with ≥20 cells were visually scored as colonies and counted.

Finally, osteogenic, adipogenic, and chondrogenic differentiations were performed. At P1 the cells were seeded in 24-well plates at a density of 5 × 10^3^ cells/cm^2^ and cultured with DMEM (Sigma Aldrich, St. Louis, MO, USA), 10% FBS (Lonza, Verviers, Belgium), 100 U/mL penicillin, 100 μg/mL streptomycin (Gibco, Invitrogen, Carlsbad, CA, USA), and 5 μg/mL plasmocin (InvivoGen, San Diego, CA, USA). For osteogenic differentiation, 50 μg/mL of ascorbic acid 2P, 7 mM of β-glycerophosphate, and 10^−7^ M of dexamethasone were added for 21 days. For adipogenic differentiation, 500 μM of isobutylmethylxanthine, 100 μM of indomethacin, 1 × 10^−6^ of dexamethasone, and 2.5 mg of insulin were added for 21 days. For chondrogenic differentiation, 5 μg/mL of insulin, 5 μg/mL of transferrin, 5 μg/mL of selenous acid, 0.1 μM of dexamethasone, 0.17 mM of ascorbic acid–2-phosphate, 1 mM of sodium pyruvate, 0.35 mM of proline and 10 μg/mL of transforming growth factor-β3 (TGF-β3) were added to the culture medium for 21 days. 

At the end of the cultures, 2% Alizarin Red S, 1.8% Oil Red O and 1% Alcian blue were used to, respectively, detect calcium deposits, lipid accumulation, and glycosaminoglycans production. 

Amniotic membranes (AM) were obtained from discarded placenta of slaughtered pregnant sheep. The timing of gestation, ranging between 2 and 3 months, was defined by considering the fetus dimensions (20–25 cm) [25]. Once the uterus wall was opened, AM in the area opposite to the umbilical cord were isolated and mechanically peeled off the chorion with the aid of a stereomicroscope. The avascular tissues were then reduced in pieces of 3–5 cm, washed in phosphate-buffered saline (PBS), and incubated in 0.25% trypsin/EDTA 200 mg/L at 37.5 °C for 20 min with continuous gentle shaking. The debris released during this digestion step was discarded. The cell suspension obtained after the enzymatic digestion was collected, filtered through a 40 µm cell filter (Sigma-Aldrich Corp., St. Louis, MO, USA), and collected into a tube containing 10% FCS (Lonza, Basel, Switzerland) to inactivate trypsin. After centrifugation, viable cells were counted by means of a hemocytometer chamber following the trypan-blue staining, to determine the number of viable cells, and characterized for their phenotype. 

The AECs were characterized by flow cytometry [26,27] for their negativity for hemopoietic markers (CD31 and CD45), positivity for both surface adhesion molecules (CD29 and CD166) and their low expression for MHC class I molecules and the absence of MHC class II (HLA-DR) antigens.

The absence of mesenchymal-derived cell contamination was also excluded by using an aliquot of freshly isolated cell in culture, and assessing, after cell adhesion, the epithelial cobblestone-like morphology and immunohistochemistry expression of epithelial and mesenchymal markers, cytokeratin-8 and alpha SMA, respectively [26]. 

Immunocytochemistry (ICC) analyses for cytokeratin-8 and alpha SMA were conducted following standardized protocols [28]. The omission of primary antibodies (Abs) was used as negative control. Flow cytometer measurement was carried out by using, as quality control, Rainbow Calibration Particles (6 peaks) and CaliBRITE beads (both from BD Biosciences). Debris was excluded from the analysis by gating on morphological parameters (lymphocyte gate); 20,000 non-debris events in the morphological gate were recorded for each sample. All antibodies were titrated under assay conditions and optimal photomultiplier (PMT) gains were established for each channel. Data were analyzed using FlowJo™ software v.10 (TreeStar, Ashland, OR, USA). Mean fluorescence intensity ratio (MFI Ratio) was calculated by dividing the MFI of positive events by the MFI of negative events.

### 2.5. Gross Evaluation 

At euthanasia (T2 and T3), both femoral condyles and tibial plateau were separated and photographed. A macroscopic score was applied to evaluate gross articular damage in the central cartilage of the medial and lateral tibial plateau, and the medial and lateral femoral condyles (4 quadrants) (Table 1) [29]. The final score was the sum of the 4 single quadrants (normal aspect of cartilage = 0; large erosions up to the subchondral bone = 16). 

### 2.6. Histology

After gross evaluation, femoral condyles, synovial membranes, and synovial fluids were sent to the laboratory. Femoral condyles were decalcified in formic acid/hydrochloric acid for about 1 month.

After decalcification, lateral condyles and synovial membranes were processed for paraffin embedding. With a semiautomated microtome (Microm H340E, Heidelberg, Germany), lateral condyles were cut on a frontal plane, while the synovial membrane on a longitudinal plane, obtaining histological sections of 5 ± 1 µm thickness. Three slides of the central portion of the condyles were stained with Safranin O/Fast Green (Sigma-Aldrich), while three slides of the synovial membranes were with hematoxylin and eosin (Sigma-Aldrich) staining. Each slide was acquired with the digital scanner Aperio ScanScope (Aperio ScanScope CS, Aperio Technologies, Leica Biosystems, Milan, Italy.) to obtain histological images.

Three slides stained with Safranin O/Fast Green of lateral condyle were scored using a modified semi-quantitative grading score: the OARSI (Osteoarthritis Research Society International) score (intact cartilage = 0; deformation = 6) [30], evaluated at magnifications of 10×, 20× and 40×.

### 2.7. Histomorphometry

Cartilage thickness (CT) and fibrillation index (FI) of lateral femoral condyles were calculated on the acquired histological images.

CT (in µm) was obtained as an average of 10 perpendicular measurements made between the upper part and the lower part of the cartilage, over the entire width of the cartilage; FI (in %) was calculated by dividing the length of the cartilage surface by the length of the area of measurement [31]. 

The histological images of synovial membrane were analyzed with the Krenn score (Table 2). This score evaluates hyperplasia/enlargement of the synovial lining cell layer, inflammatory infiltration, and activation of synovial stroma/pannus formation (no synovitis = 0; high degree of synovitis = 9) [32].

### 2.8. Immunohistochemistry

The other 3 slides of the lateral femoral condyles were immunostained for COLL II, COLL I, MMP13 and IL1β, using primary antibodies (anti-COLL I, COLL II, MMP13 and IL1β). 

The amount of COLL II, COLL I, MMP13 and IL1β (in %) was calculated as the ratio between immunopositively stained areas and the total region of interest areas. 

### 2.9. Biochemistry of the Synovial Fluid 

Biochemical evaluations of the synovial fluid were performed through the quantification of pro-inflammatory cytokines, usually present in an OA knee. IL1β, Cross Linked C Telopeptide of Type II Collagen (CTX2), TNFα, IL6 and Prostaglandin E2 (PGE2) were quantified with ELISA kits, according to manufacturer indications (ABclonal Technology, Woburn, MA, USA). From collection to evaluations, synovial fluids were maintained at −80 °C. 

### 2.10. Statistical Analysis

The power analysis considered: (1) the factor ‘treatments’ had 4 levels (AEC, ADSC, SVF and NaCl) and the factor ‘experimental time’ had 2 levels (3 and 6 months); (2) treatments were placed in both knee joints by using a combination, C (4.2). According to these parameters, a minimum number of 24 sheep were required, corresponding to n = 6 knee joints for treatment, and experimental time with a power of 80% and a *p*-value < 0.05. A total of 48 knee joints were injected. Statistical evaluation was performed using SPSS/PC + StatisticsTM 25.0 software (SPSS Inc., Chicago, IL, USA). Data were reported as mean ± standard deviation at a significance level of *p* < 0.05. The data did not show a normal distribution and homogeneity of the variance (Levene test), and, therefore, a non-parametric analysis was performed using Kruskal–Wallis; Mann–Whitney U test was performed to compare the different treatment groups. Student’s test was used to analyze the parameters evaluated over time (3 and 6 months).

For the analysis of the OARSI data, an ordinal regression model (cumulative link model, CLM) was used for repeated measures (section) and the factors considered were the ‘Treatment’ (4 levels: NaCl, ADSC, AEC and SVF) and ‘Experimental time’ (2 levels: 3 and 6 months). The analysis showed no interactions of the two factors on the OARSI data (Chi^2^ = 3.41, *p* = 0.333). Instead, there were effects on the two factors: ‘Treatment’, Chi^2^ = 43.3, *p* < 0.0005; ‘Experimental time’, Chi^2^ = 70.7, *p* < 0.0005. The variance between repeated measures contributed minimally to the variability of the variance of the model Var = 1.87 × 10^−8^.

## 3. Results

### 3.1. ADSCs and AECs Characterization

**Figure 2:**
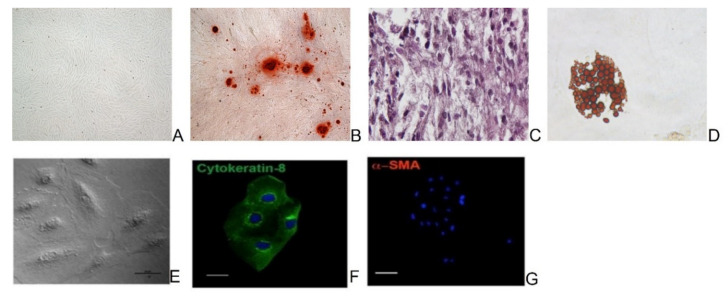
Microscopic images of sheep ADSCs cultured in normal (**A**), osteogenic (**B**), chondogenic (**C**), and adipogenic (**D**) media for the three lineages differentiation test. Magnification ×4. Representative images of in vitro cultured AECs displaying a polyhedral phenotype (phase contrast) (**E**), cytokeratin 8 positivity (green color) (**F**), and alpha SMA negativity (**G**). Nuclei were counterstained with DAPI (blue color). Scale Bars: 10 μm for phase contrast and cytokeratin 8 images, and 50 μm for alpha SMA image.

ADSCs differentiated into osteogenic, chondrogenic and adipogenic lineages as indicated in Figure 2A–D. Furthermore, the cells showed high CD44, CD73, CD90 and CD105, and weak CD31, CD45 and CD34 markers. 

AECs showed, in culture, a typical polyhedral phenotype characterized by the cytokeratin 8 epithelial marker expression and the absence of the mesenchymal marker alpha SMA (Figure 2E–G). They did not display CD31 and CD45, showed a low expression of MHC class I, and the absence of MHC class II molecules. On the contrary, the cells expressed the surface adhesion molecules CD29 and CD166. 

### 3.2. Clinical and Gross Evaluations

The animals well tolerated the anesthesia, surgery, and all types of treatments. No adverse or side effects were observed over time.

At the time of the explant, no signs of necrosis or degeneration were observed in any animal and at any experimental time. No animals were excluded from the analyses.

At gross evaluation, the lateral compartment of the femoral condyles of all animals was the most affected by the typical degeneration signs of OA, as also documented in the literature [24]. For this reason, histology, histomorphometry and immunohistochemistry were performed on the lateral condyles.

Large erosion up to the subchondral bone was not observed in any group at any experimental time, but at 6 months small erosions up to the subchondral bone, less than 5 mm in size, were observed for ADSCs and NaCl groups. In the other two groups, superficial fibrillation and fissures were present at both 3 and 6 months.

**Figure 3:**
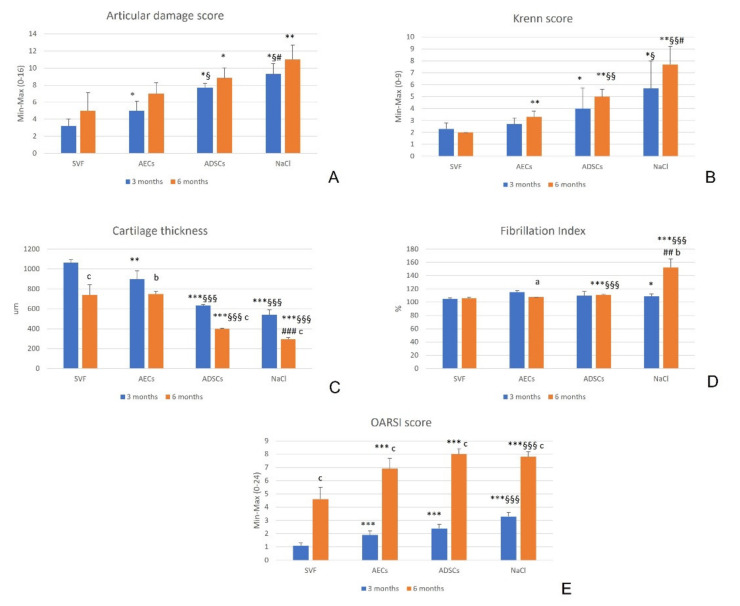
Mean and standard deviation (SD) of macroscopic score (**A**), Krenn score (**B**), cartilage thickness (CT) (µm) (**C**), fibrillation index (FI) (%) (**D**), and OARSI score (**E**), for all treatment groups: SVF, AECs, ADSCs and NaCl, at 3 and 6 months. N = 6. *, *p* < 0.05; **, *p* < 0.005; ***, *p* < 0.0005: SVF vs. AECs, ADSCs and NaCl. §, *p* < 0.05; §§, *p* < 0.005; §§§, *p* < 0.0005: AECs vs. ADSCs and NaCl. #, *p* < 0.05; ##, *p* < 0.005; ###, *p* < 0.0005: ADSCs vs. NaCl. a, *p* < 0.05; b, *p* < 0.005; c, *p* < 0.0005: 6 months vs. 3 months.

At 3 months, SVF significantly reduced the articular damage score (*p* = 0.04, *p* = 0.012, *p* = 0.04, for AECs, ADSCs and NaCl, respectively). AECs had a more significantly reduced score than ADSCs (*p* = 0.012) and NaCl (*p* = 0.03), and ADSCs than NaCl (*p* = 0.03). At 6 months, SVF showed significantly lower values than ADSCs (*p* = 0.023) and NaCl (*p* = 0.001). No significant difference was observed between the experimental times for all treatment groups (*p* = 0.09, *p* = 0.15, *p* = 0.51 and *p* = 0.44, for SVF, AECs, ADSCs and NaCl, respectively) (Figure 3A).

### 3.3. Synovial Membrane 

**Figure 4:**
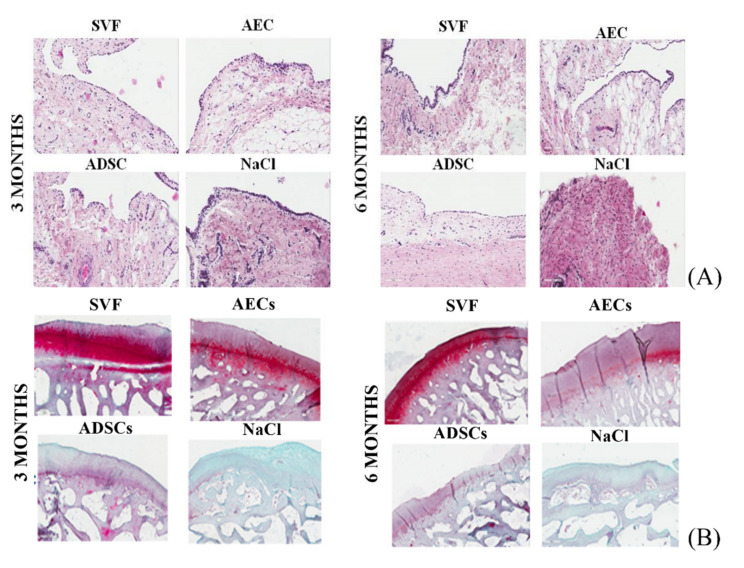
(**A**) Histological images of synovial membrane at 4× magnification. Hematoxylin and eosin staining. (**B**) Histological images of lateral femoral condyle cartilage at 4× magnification. Safranin O–Fast Green staining. Scale bar = 500 µm.

At 3 months, the synovial membrane of ADSCs and NaCl groups presented enlargement of the cell layer of the synovial lining, with a moderate increase in cellularity in comparison with the other two groups treated with SVF and AECs. At 6 months, the worst aspect of the synovium was observed in the NaCl group, with several cell layers of the synovial lining and a moderate increase in cells with multinucleated cells (Figure 4A).

The Krenn score at 3 months was significantly lower in the SVF group, than in the ADSCs (*p* = 0.04) and NaCl (*p* = 0.019) groups. The score in the AEC group was better than in the NaCl group (*p* = 0.032). At 6 months, the SVF group showed the lowest values (*p* = 0.002), the AEC group had lower results than the ADSCs (*p* = 0.004) and NaCl (*p* = 0.003) groups, and ADSCs showed a significantly reduced Krenn score in comparison with NaCl (*p* = 0.012). No significant difference was observed over time for all treatment groups (*p* = 0.15, *p*=0.05, *p*=0.21 and *p* = 0.10 for SVF, AECs, ADSCs and NaCl, respectively) (Figure 3B). 

### 3.4. Cartilage 

As shown in Figure 4B, no severe signs of OA were observed at either 3 or 6 months. None of the specimens showed abrasion of the cartilage up to exposure of the subchondral bone in any group.

In the worst group (treated with NaCl), there was a reduction in the cartilage staining throughout the thickness, thinning of the CT and increased fibrillation, both at 3 and at 6 months. Additionally, in the ADSC group, cartilage staining was less intense than in the other two groups with reduced CT and increased fibrillation, which worsened at 6 months. It was also noted that CT decreased over time in the SVF group and fibrillation increased over time in the group treated with AECs. 

Regarding histomorphometry evaluations, at 3 months, the SVF group showed significantly higher CT than AECs (*p* = 0.002), ADSCs and NaCl (*p* < 0.0001) groups. Additionally, AECs induced significantly higher CT than ADSCs and NaCl (*p* < 0.0001). At 6 months, SVF and AECs had significantly increased CT compared with ADSCs and NaCl (*p* < 0.0001), and ADSCs had better results than NaCl (*p* < 0.0001). CT decreased over time for all treatments (*p* < 0.0001 for SVF, ADSCs and NaCl; *p* = 0.001 for AECs) (Figure 3C). 

At 3 months, in the SVF group, FI values were significantly lower than in the NaCl group (*p* = 0.008). At 6 months, SVF and AECs induced significantly lower FI than ADSCs and NaCl (*p* < 0.0001), and ADSCs values were significantly lower than the NaCl group (*p* = 0.001). Over time, the FI of AECs decreased (*p* = 0.02), while the FI of NaCl increased (*p* = 0.001) (Figure 3D). 

At 3 and 6 months, the OARSI score significantly improved with SVF in comparison with all other treatments (*p* < 0.0005), and with AECs in comparison with NaCl (*p* = 0.0003). Over time, all values worsened in all groups (*p* < 0.0003) (Figure 3E).

**Figure 5:**
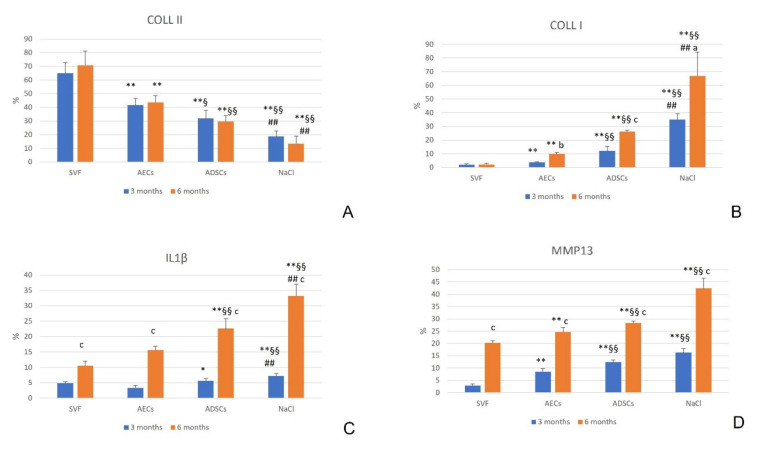
Mean and standard deviation (SD) of immunostaining quantification (%) of Collagen II (COLL II) (**A**), Collagen I (COLL I) (**B**), Interleukin 1β (IL1β) (**C**), and Metalloproteinase 13 (MMP13) (**D**), for all treatment groups: SVF, AECs, ADSCs and NaCl, at 3 and 6 months. N = 6. *, *p* < 0.05; **, *p* < 0.005: SVF vs. AECs, ADSCs and NaCl. §, *p* < 0.05; §§, *p* < 0.005: AECs vs. ADSCs and NaCl. ##, *p* < 0.005: ADSCs vs. NaCl. a, *p* < 0.05; b, *p* < 0.005; c, *p* < 0.0005: 6 months vs. 3 months.

At 3 and 6 months, COLL II was significantly higher in the SVF group than in the other groups (*p* = 0.004), in the AEC group than in the ADSCs and NaCl groups (*p* = 0.02 for ADSCs at 3 months, *p* = 0.004 for NaCl at 3 months, and for ADSCs and NaCl at 6 months), and in the ADSC group than in the NaCl group (*p* = 0.004). No significant difference was observed among experimental times (*p* = 0.28, *p* = 0.50, *p* = 0.43 and *p* = 0.07 for SVF, AECs, ADSCs and NaCl, respectively) (Figure 5A).

COLL I, at 3 and 6 months, was significantly lower in the SVF group than in the other groups (*p* = 0.004), and in the AEC group than in the ADSCs and NaCl groups (*p* = 0.004). In addition, ADSCs significantly decreased COLL I compared with NaCl (*p* = 0.004). Over time, COLL I significantly increased in the AECs (*p* = 0.003), ADSCs (*p* < 0.0001) and NaCl (*p* = 0.006) groups (Figure 5B).

At 3 months, SVF (*p* = 0.03) and AECs (*p* = 0.006) significantly reduced IL1β percentage more than ADSCs and NaCl, which showed the highest values (*p* = 0.004). At 6 months, the SVF and AEC groups showed a significantly lower percentage than the ADSCs and NaCl (*p* = 0.004) groups, and the ADSCs group was lower in comparison with the NaCl group (*p* = 0.004). Over time, IL1β percentage increased in all treatments (*p* < 0.0001) (Figure 5C). 

MMP13, at 3 and 6 months, was significantly reduced by SVF versus the other groups (*p* = 0.004), and the AECs group showed a lower percentage than the ADSCs and NaCl groups (*p* = 0.004). All treatments significantly increased MMP13 over time (*p* < 0.0001) (Figure 5D). 

### 3.5. Synovial Fluid

**Figure 6:**
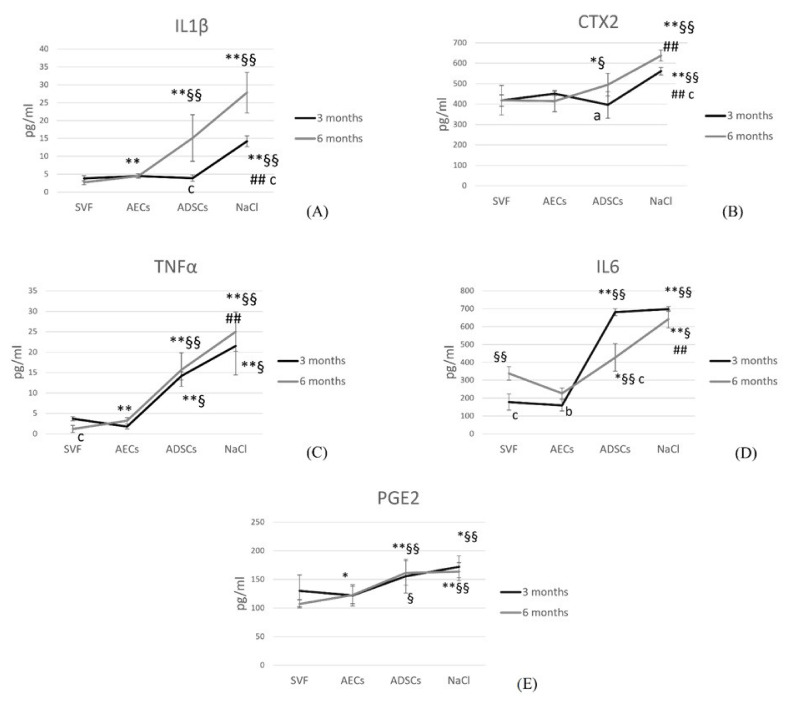
Line graphs of pro-inflammatory cytokine quantification (pg/mL) in the synovial fluid: IL1β (**A**), CTX2 (**B**), TNFα (**C**), IL6 (**D**), PGE2 (**E**), in all treatment groups: SVF, AECs, ADSCs and NaCl at 3 (black line) and 6 (grey line) months. N = 6. *, *p* < 0.05; **, *p* < 0.005: SVF vs. AECs, ADSCs and NaCl; §, *p* < 0.05; §§, *p* < 0.005: AECs vs. ADSCs and NaCl; ##, *p* < 0.005: ADSCs vs. NaCl. a, *p* < 0.05; b, *p* < 0.005; c, *p* < 0.0005: 6 mo vs. 3 mo.

At 3 months, IL1β was significantly reduced with SVF, AECs and ADSCs in comparison with NaCl (*p* = 0.001). At 6 months, IL1β was significantly reduced by SVF compared with AECs, ADSCs and NaCl (*p* = 0.001), and by AECs compared with ADSCs and NaCl (*p* = 0.001). Over time, ADSCs and NaCl significantly increased IL1β (*p* < 0.0001) (Figure 6A). 

At 3 and 6 months, CTX2 was significantly lower in SVF, AECs and ADSC groups than in the NaCl group (*p* = 0.001). In addition, at 6 months, SVF (*p* = 0.03) and AECs (*p* = 0.04) significantly decreased CTX2 compared with ADSCs. ADSCs (*p* = 0.006) and NaCl (*p* < 0.0001) significantly increased CTX2 over time (Figure 6B). 

At 3 months, TNFα significantly decreased in SVF and AECs groups in comparison with ADSCs and NaCl groups (*p* = 0.001). At 6 months, SVF showed the lowest amount of TNFα (*p* = 0.001), AECs significantly decreased it compared with ADSCs and NaCl (*p* = 0.001), and ADSCs significantly reduced its values compared with NaCl (*p* = 0.002). Over time, SVF significantly decreased TNFα (*p* < 0.0001) (Figure 6C).

At 3 months, IL6 was significantly lower in SVF and AECs groups than in ADSCs and NaCl groups (*p* = 0.001). At 6 months, AECs significantly reduced IL6 compared with SVF, ADSCs and NaCl (*p* = 0.001). SVF significantly reduced IL6 versus ADSCs (*p* = 0.016) and NaCl (*p* = 0.001), and finally, also, ADSCs significantly reduced IL6 versus NaCl (*p* = 0.001). Over time, SVF (*p* < 0.0001) and AECs (*p* = 0.001) significantly increased IL6, and ADSCs significantly lowered it (*p* < 0.0001) (Figure 6D). 

PGE2 was significantly reduced by SVF in comparison with NaCl (*p* = 0.009), and by AECs in comparison with ADSCs (*p* = 0.03) and NaCl (*p* = 0.001), at 3 months. At 6 months, SVF significantly reduced PGE2 compared with AECs, ADSCs (*p* = 0.046) and NaCl (*p* = 0.001), in addition to AECs versus ADSCs (*p* = 0.002) and NaCl (*p* = 0.001). No statistical difference was observed over time for all treatments (*p* = 0.055, *p* = 0.95, *p* = 0.65, *p* = 0.36 for SVF, AECs, ADSCs and NaCl, respectively) (Figure 6E).

## 4. Discussion

The main finding of this study is the understanding of the potential of different biological approaches to address OA. While all strategies performed better than the saline control, this study demonstrated, by a direct comparison in the same large animal model, the more favorable outcome of SVF over ADSCs and AECs, in addition to the potential of AECs to provide positive results without the need for the invasive step of autologous tissue harvesting. These results represent an important step forward in defining the most suitable strategy for the management of this challenging pathology.

The resolution of OA in an early phase, through minimally invasive orthobiological solutions, would be important to return to daily and sport activities of the affected patients, with important improvements in the quality of life, delaying the need for more invasive prosthesis solutions. To this aim, the present in vivo study compared and evaluated, in the same large animal model, the potential of three different biological injective treatments in counteracting the spread of mild OA course at two experimental times. The main innovative aspects of this study are the comparison of three injective biological treatments in the same large size animal model; the use of AECs in vivo for OA treatments; and the evaluation of both structural and inflammatory fronts of the pathology at 6 months. Overall, the present study showed better results with SVF, followed by AECs and ADSCs in terms of macroscopic aspects of the knee, cartilage structure of femoral condyles, and pro-inflammatory cytokine production in the synovial fluid. 

The potential of ADSCs in OA pathology has been known for years, in both in vivo studies and clinical trials [7,8,9,33,34] and the results of the literature were confirmed by those of the present study. In comparison with the control (NaCl), culture expanded ADSCs significantly ameliorated the macroscopic aspect of the articular cartilage, Krenn score, CT, FI, COLL II, COLL I, IL1β, and all pro-inflammatory cytokines of the synovial fluid, except for PGE2. However, the clinical use of ADSCs could be complicated by donor site morbidity, ageing or disease of the donor, the necessity of a previous in vitro expansion to obtain a large cell number able to produce a clinical effect and the associated risks of cell transformation or infection [35].

To avoid the risks related to ADSCs, other two cell sources, SVF and AECs, were investigated, and the results were compared with those of ADSCs. The potential of minimal adipose tissue manipulation over cell expansion was recently documented in a rabbit OA model [36], and in a preliminary study [37] in sheep comparing the effects of the same three cellular treatments. In this last study, the results at 3 months showed no severe degeneration, and improvements in terms of macroscopic cartilage surface and reduction in pro-inflammatory cytokines in the synovial fluid, by all treatments. In addition, SVF highlighted the best performance while ADSCs were the worst [37]. 

The superiority of SVF treatment over the other types was confirmed at 6 months, both in terms of cartilage regeneration and in counteracting the inflammatory microenvironment. The AEC treatment followed SVF and showed superiority over ADSC. More precisely, SVF and AECs had a similar trend in FI, IL1β percentage in cartilage and CTX2 values in the synovial fluid, and both made improvements in all the parameters analyzed, in comparison with ADSCs and NaCl. Between SVF and AECs, the first treatment showed more improvements, in some aspects, in comparison with the second treatment: macroscopic score and CT at 3 months, Krenn score at 6 months, and COLL II, COLL I, MMP13, pro-inflammatory cytokines production, except for CTX2, and OARSI score, at 3 and 6 months. 

These results regarding SVF find support in preclinical and clinical literature data: SVF reduced COLL X and increased stromal cell-derived factor 1 (SDF1) production more than ADSCs or hyaluronic acid alone [6], improved joint regeneration [10], was safe, resolved pain, and increased physical function in comparison with hyaluronic acid, also up to 18 months [11], in combination with PRP [38,39], microfracture [12] or arthroscopic debridement [13]. 

No study evaluated AECs in OA. AECs could be considered a valid alternative to SVF or ADSCs because they differentiate into three lineages, express mesenchymal and embryonic stem cell markers, show a non-tumorigenic phenotype and have a high yield. In addition, AECs replace embryonic stem cells, which has a clear impact on ethical matters [16]. AECs have been used for other types of musculoskeletal tissue regeneration with success, such as bone and tendon: AECs ex vivo repair human cartilage defects and were able to chondrogenically differentiate in micromasses [22,23]. 

For the direct comparison of these cell-based treatments in OA, this study employed an in vivo large animal model. Even if there is an increasing trend to replace animal models with in vitro alternatives, following the 3R principles, the use of large animal models is still required to finalize the translation between preclinical research and patient care. Since the injection of cells into a living organism can drastically change cellular and host response, this comparative in vivo study is deemed fundamental before considering a clinical translation. In this light, the sheep was the animal of choice for this study, due to its similarity with human knee anatomy, in addition to terms of advancement of OA pathology and pathological signs in cartilage and subchondral bone [40]. Meniscus also shows similarity in vascularization, cellular composition, collagen structure and size, compared to human ones [41]. In addition, to reduce the number of animals, as required by 3R principles, we decided to inject the coupled treatments as indicated in 2.2 paragraph of the “Materials and Methods” section, even if this might lead to bias due to the influence of animal-to-animal variability. Several different surgical techniques can be used to in vivo induce OA at different degrees of severity. Meniscectomy is the technique most used in large animal models. It increases OA risk by a factor of six [42], because it induces anomalies in abduction and translation of the tibia, leading to an abnormal stress between femur and tibia and cartilage damage in the central weight bearing zone [43]. After meniscectomy, the classical signs of OA are present, such as articular space narrowing, osteophytes, and cartilage damage [44,45]. Bilateral meniscectomy is preferably used instead of monolateral, to reduce the number of animals exposed to such procedures, and no adverse events, associated with bilateral options, have previously been reported in the literature. In addition, meniscectomy is preferred to the anterior cruciate ligament transaction (ACLT) procedure because the latter induces a severe OA. As indicated by Delling et al., following 6 weeks after bilateral lateral meniscectomy, mild to moderate OA is developed [46].

## 5. Conclusions

Significant improvements in joint structure and inflammation were observed after 3 and 6 months from a single i.a. injection of AECs and SVF in a sheep preclinical model of OA. All three treatments improved outcome measures (which included macroscopic tissue evaluation, Krenn scores for the synovium, inflammatory cytokines in synovial fluid, in addition to cartilage thickness and fibrillation thickness) compared with the saline solution (CTR), with SVF treatment being superior to AECs which, in turn, was superior to ADSCs.

In the present study, a single i.a. dose of treatment was adopted to observe if one dose may be sufficient in view of clinical translatability. However, from 3 to 6 months, CT decreased and COLL I, IL1β, MMP13 and IL6 of the synovial fluid and OARSI score increased, for all treatments. Even if the parameters that worsened were few, it could be expected to increase the number of doses in the future; for example, it could be considered to plan a second dose, 3 months after the first one, to evaluate if the results further ameliorate over time. An amount of 2.5 × 10^6^/mL cells was chosen in this study. Considering the enormous variability in the literature studies of the cell amount injected, and considering that many cells are injected together with scaffolds or other therapeutic substances, we chose this order of magnitude based on those employed in clinical trials [47]. Still, it is possible that different doses might lead to different results. Despite these limitations, the study findings offer new important insight in this rapidly evolving field. 

## Figures and Tables

**Table 1:** Gross articular damage score, assessed in the central cartilage of the medial and lateral tibial condyles, medial and lateral femoral condyles (4 quadrants). Each total score is the sum of the score of each quadrant. Minimum = 0; maximum = 16.

Gross Articular Damage Score	
**Normal**	0
Fibrillations and fissures	2
Small erosions up to the subchondral bone (<5 mm diameter)	3
Large erosions up to the subchondral bone (>5 mm diameter)	4
**Sum**	**0–16**

**Table 2:** Krenn score for the synovial membrane. Each total score is the sum of the subscores. Minimum = 0; maximum = 9.

Enlargement of the Cell Layer of the Synovial Lining	
1 layer	0
2–3 layers	1
4–5 layers, few multinucleated cells	2
5 layers, the layer can be ulcerated and presence of multinucleated cells	3
**Cell density of synovial stroma**	
Normal cellularity	0
Cellularity slightly increased	1
Cellularity moderately increased, multinucleated cells may be present	2
Cellularity greatly increased, giant multinucleated cells, cloth formation and rheumatoid granuloma	3
**Inflammatory infiltrate**	
No inflammatory infiltrate	0
Few perivascular lymphocytes or plasma cells	1
Numerous lymphocytes or plasma cells, which form follicular aggregates	2
Dense inflammatory infiltrate or numerous follicular aggregates	3
**Sum**	
No synovitis	0 or 1
Low degree of synovitis	2–4
High degree of synovitis	5–9

## Data Availability

The raw data supporting the conclusions of this article will be made available by the authors, upon reasonable request, without undue reservation.
